# Activation of AK005401 aggravates acute ischemia/reperfusion mediated hippocampal injury by directly targeting YY1/FGF21

**DOI:** 10.18632/aging.102106

**Published:** 2019-07-23

**Authors:** Hongzhi Wan, Ying Yang, Miao Li, Xin Liu, Yeying Sun, Kexin Wang, Chunxiang Zhang, Qingyin Zheng, Chaoyun Wang

**Affiliations:** 1 School of Pharmacy, Binzhou Medical University, Yantai, P. R. China; 2 Third class of senior high school, NO.2 Middle School of Yantai Shandong, Yantai, P. R. China; 3 Department of Biomedical Engineering and Department of Medicine, School of Medicine, University of Alabama at Birmingham, Birmingham, AL 35233, USA; 4 Department of Otolaryngology-Head and Neck Surgery, Case Western Reserve University, Cleveland, OH 44106, USA; 5 Hearing and Speech Institute, Binzhou Medical University, Yantai, P. R. China

**Keywords:** Ak005401, ischemia/reperfusion, antioxidant capacity, mitochondria dysfunction, fibroblast growth factor 21

## Abstract

Ischemia exerts a negative impact on mitochondrial function, which ultimately results in neuronal damage via alterations in gene transcription and protein expression. Long non- coding RNAs (LncRNAs) play pivotal roles in the regulation of target protein expression and gene transcription. In the present study, we observed the effect of an unclassical LncRNA AK005401on ischemia/reperfusion (I/R) ischemia-mediated hippocampal injury and investigated the regulatory role of fibroblast growth factor 21 (FGF21) and Yin Yang 1 (YY1). C57Black/6 mice were subjected to I/R using the bilateral common carotid clip reperfusion method, and AK005401 siRNA oligos were administered via intracerebroventricular injection. HT22 cells were used to establish a model of oxygen-glucose deprivation/reoxygenation (OGD/R). We observed pathological morphology and mitochondrial structure. Neuronal apoptosis was evident. Cell activity, cell respiration, FGF21, YY1, and antioxidant capacity were evaluated. I/R or OGD/R significantly increased the expressions of AK005401and YY1 and decreased FGF21expression, which further attenuated the activation of PI3K/Akt, promoted reactive oxygen species (ROS) generation, and then caused mitochondria dysfunction and cell apoptosis, which were reversed by AK005401 siRNA oligos and were aggravated by overexpression of AK005401 and YY1. We conclude that AK005401/YY1/FGF21 signaling pathway has an important role in I/R-mediated hippocampal injury.

## INTRODUCTION

Stroke seriously affects the health of people in the world, and frequently causes death or long-term disability [1]. In patients with cerebral ischemia—which accounts for approximately 80% of stroke cases—the disruption of blood flow to the brain results in neuronal injury and stimulates the generation of reactive oxygen species (ROS) [2, 3]. In addition, subsequent restoration of blood flow and reoxygenation may exacerbate damage to neuronal tissues [4].

Mitochondria, as important bioenergy organelles, not only synthesize Adenosine triphosphate (ATP) but also produce ROS during the electron transfer stages of the respiratory chain. Although this process is tightly regulated under physiological conditions, ischemia causes mitochondrial dysfunction, allowing electrons to escape from the mitochondrial respiratory chain and interact with oxygen molecules to form ROS [5]. Ischemia and reperfusion inhibit the activity of endogenous antioxidant enzymes and promote the overproduction of ROS [6–8]. These ROS then damage the mitochondrial membrane, facilitating the release of cytochrome C and promoting the activation of caspase 3, ultimately leading to cellular apoptosis [9–11].

FGF21 is secreted into the circulation and can travel to sites distal from its origin, acting predominantly via an endocrine mechanism [12]. Previous studies have revealed that fibroblast growth factor 21 (FGF21), which exerts pleiotropic effects on the regulation of glucose metabolism [13], also influences mitochondrial function [14, 15]. Planavila et al demonstrated that FGF21 regulates the expression of genes involved in antioxidant pathways, thus preventing the production of ROS in cardiac cells [16]. Additional studies have indicated that FGF21 attenuates diabetes-induced renal oxidative stress, inflammation, apoptosis, and lipid/collagen accumulation via upregulation of nuclear factor like(erythroid-derived 2) (Nrf2) and activation of the PI3K/Akt pathway [17].

Yin Yang 1 (YY1) is a ubiquitous transcription factor that interacts with histone acetyltransferases and deacetylases to activate or suppress gene transcription, although recent studies have indicated that it is also involved in the regulation of inflammation [18, 19]. Overexpression of YY1 increases the expression of *Bax* mRNA and protein by binding to the *Bax* promoter [20]. Srivastava et al demonstrated that YY1 reduces Cu/Zn-superoxide dismutase (Cu/Zn–SOD) expression and increases ROS generation in the heart [21].

**Figure 1:**
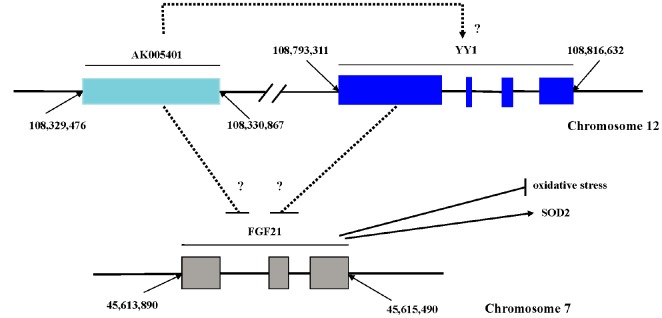
**Sequence site and possible relationships of AK005401, FGF21 and YY1.** AK005401 is 1392 bp non-coding RNA sequence, which derived from the gene sequence (from 108329474 to 108330865) located on chromosome 12. FGF21 is 947 bp including three exon sequences (from 45,613,890 to 45,615,490) located on chromosome 7. YY1 located on chromosome 12 as like as AK005401 is from 108,793,311 to 108,816,632.

Long non coding RNAs (LncRNAs) can be classified into sense, antisense, intronic, bidirectional, and intergenic forms according to their relationship with adjacent protein-coding genes [22], which play key roles in various cellular contexts under both physiological and pathological conditions and participates in various biological processes, such as critical regulators of microRNAs [23, 24], cell cycle control, cell differentiation and apoptosis, transcriptional and translational control, epigenetic silencing, and splicing regulation [25–28]. AK005401 is a 1,392-base pair (bp) noncoding RNA sequence derived from a gene sequence (from 108329474 to 108330865) located on chromosome 12 (Figure 1). In our experiment, we discovered that AK005401 is involved in the regulation of cerebral damage mediated by ischemia/reperfusion (I/R). In the present study, we aimed to determine whether AK005401 is involved in the promotion of I/R-mediated brain injury and the reduction of antioxidant capacity and whether the mechanisms underlying these alterations are associated with changes in YY1 or FGF21 expression.

## RESULTS

### Effect of AK005401 on I/R-induced neuronal damage

**Figure 2:**
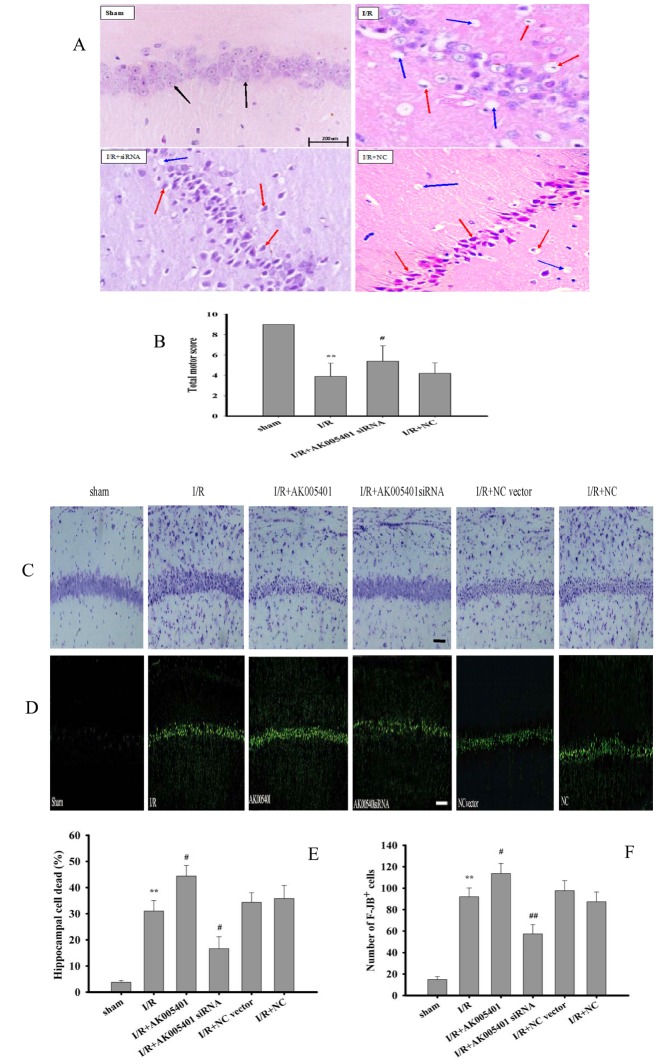
**Effect of AK005401on ischemia/reperfusion (I/R) induced mice hippocampus pathomorphology and motor score.** The mice were divided into four groups: sham, I/R, AKsiRNA, and NC. In addition, AK005401 overexpression vector group and NC vector group were added. After reperfusion for 24 hours, the motor score of each mouse was obtained according to the neurological test method. The brains were fixed, embedded in paraffin, cut into 4-μm-thick sections, and stained with HE, cresyl violet and FJB. (**A**) The neurons in the CA1 hippocampus stained with HE were observed by light microscopy. Light microscopy shows normal neuronal cells (black solid line arrows), pathological neurons with shrunken cytoplasm, damaged nuclei (red solid line arrows), and vacuolization (blue solid line arrows). (**B**) Total motor scores in all groups were showed (n = 15). (**C**) The neurons stained with cresyl violet were observed by light microscopy. (**D**) The neurons stained with FJB were observed by light microscopy. (**E**) The percentage of cell deaths was analyzed (n=3). (**F**) F-JB^+^ cells were counted (n=3). Data were presented as mean±SD. One-way ANOVA test was used to determine statistical significance. **P < 0.01 vs. sham group, ^#^*P* < 0.05 vs. I/R group.

As shown in Figure 2, motor scores in the I/R group were significantly lower than those in the sham group (*P* < 0.01), although such deficits were attenuated by treatment with AK005401 siRNA (Figure 2B, *P* < 0.05). We then assessed the histopathological changes of neurons, in hippocampus tissues after treatment with I/R, inhibition or overexpression of AK005401 expression. No significant morphological changes were observed in neurons of the sham group. However, neurons in the I/R and NC siRNA oligos groups exhibited significant degeneration, swelling, atrophy, vacuolization and high mortality (*P* < 0.01), which were aggravated by Overexpression of AK005401(*P* < 0.05). Treatment with AK005401siRNA significantly attenuated such morphological changes and decreased cell death (*P* < 0.05 or *P* < 0.01).

### Effect of AK005401 on OGD/R-mediated cell injury

**Figure 3:**
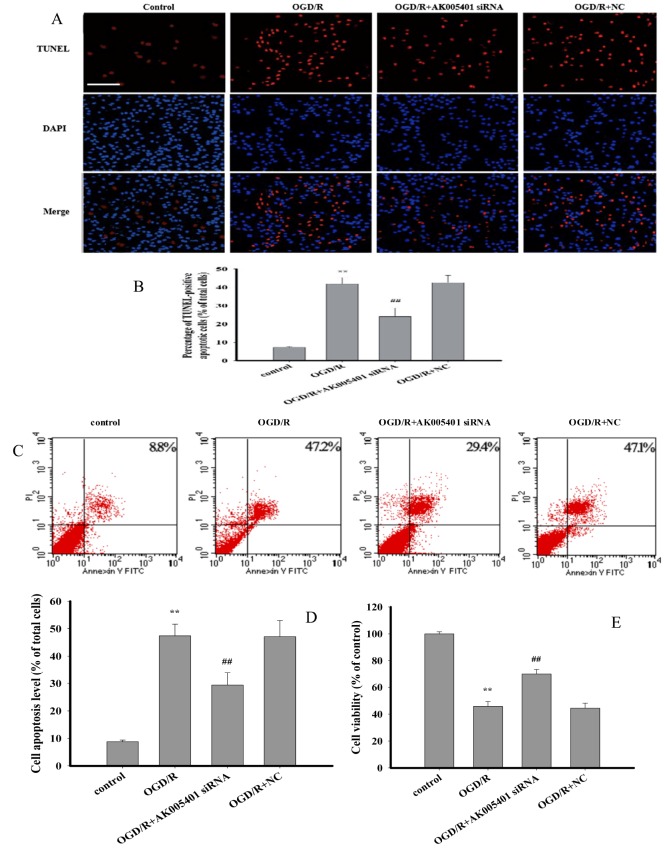
**Effect of AK005401 on cell viability and apoptosis.** HT22 cells were divided in four groups: control, OGD/R, AK005401siRNA, and NC. Cell viability was detected by MTT method. Cell apoptosis of all groups was observed by applying TUNEL apoptosis assay kit and AnnexinV/PI apoptosis assay kit, respectively. (**A**) apoptotic cells were visualized by TUNEL staining (red). Nuclei were counterstained with 4′,6-diamidino-2-phenylindole (DAPI, blue). (**B**) TUNEL positive apoptotic cells were counted as a percentage of the total number of cells. (**C**) Cell activities were evaluated using AnnexinV/PI apoptosis assay kit. (**D**) Apoptotic cells obtained from AnnexinV/PI method were counted as a percentage of the total number of cells. (**E**) Cell viabilities of all groups were measured by using MTT methods. Data were presented as mean±SD (n = 3 for apoptosis, n = 10 for cell viability). One-way ANOVA test was used to determine statistical significance. **P < 0.01 vs. control group, ^##^*P* < 0.01 vs. OGD/R group.

As shown in Figure 3, oxygen-glucose deprivation/reoxygenation (OGD/R) significantly reduced cell viability, increased the number of TUNEL-positive apoptotic cells, and enhanced the percentage of viable apoptotic cells, relative to findings observed in control mice (*P* < 0.01). However, treatment with AK005401 siRNA effectively attenuated OGD/R-mediated cell injury by increasing cell activity and reducing the apoptosis of exposed cells (*P* < 0.01).

**Figure 4:**
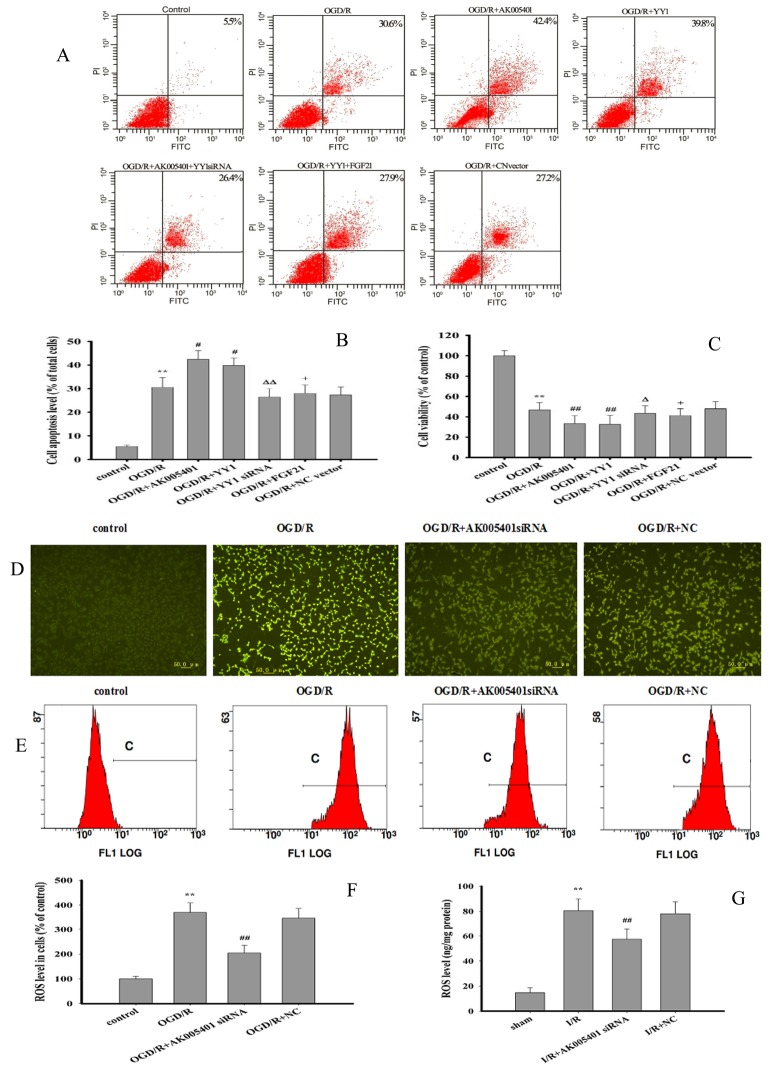
**Effect of overexpression of AK005401and YY1 on cell viability and apoptosis and effect of AK005401 on ROS generation.** Mice and HT22 cells were used to establish I/R model and OGD/R model, respectively. Cells were collected and seeded in a 96-well plate or 6-well plate and divided into seven groups as follows: control, OGD/R, OGD/R+AK005401 (OGD/R+ AK005401 overexpression vector), OGD/R+YY1 siRNA (OGD/R + AK005401 overexpression vector +YY1 siRNA), OGD/R+YY1 (OGD/R+ YY1 overexpression vector), OGD/R+FGF21 (OGD/R + YY1 overexpression vector +FGF21 overexpression vector) and OGD/R+NC vector(OGD/R+ negative control vector) groups. (**A**) Cell activities were evaluated using AnnexinV/PI apoptosis assay kit. (**B**) Apoptotic cells obtained from AnnexinV/PI method were counted as a percentage of the total number of cells. (**C**) Cell viability was detected by MTT method. ROS levels in all groups were detected using fluorescent microscope and flow cytometry. (**D**) ROS levels were observed using fluorescent microscope after treatment with DCFH-DA. (**E**) ROS production was analyzed by applying flow cytometry. (**F**) Effects of AK005401 on ROS levels in HT22 cells (n = 3 experiments). (**G**) the ROS contents in hippocampus tissues were measured at 450 nm according to the procedures described by ROS ELISA assay kits. Data were presented as mean±SD (n = 10 in hippocampus tissues, or n = 3 in cells). One-way ANOVA test was used to determine statistical significance. **P < 0.01 vs. sham group or control group, #P < 0.05 or ##P < 0.01 vs. I/R group or OGD/R group, ΔP < 0.05 or ΔΔP < 0.01 vs.OGD/R+AK005401 group, +P < 0.05 vs.OGD/R+YY1 group.

As shown in Figure 4A–4C, overexpression of AK005401 or YY1 remarkably increased the percentage of viable apoptotic cells and reduced cell viability (*P* < 0.05 or *P* < 0.01). Treatment with YY1 siRNA or FGF21 overexpression effectively alleviated AK005401 or YY1 overexpression mediated cell injury by enhancing cell activity and reducing apoptosis, respectively (*P* < 0.05 or *P* < 0.01).

### Effect of AK005401 on antioxidant capacity and ROS level

**Figure 5:**
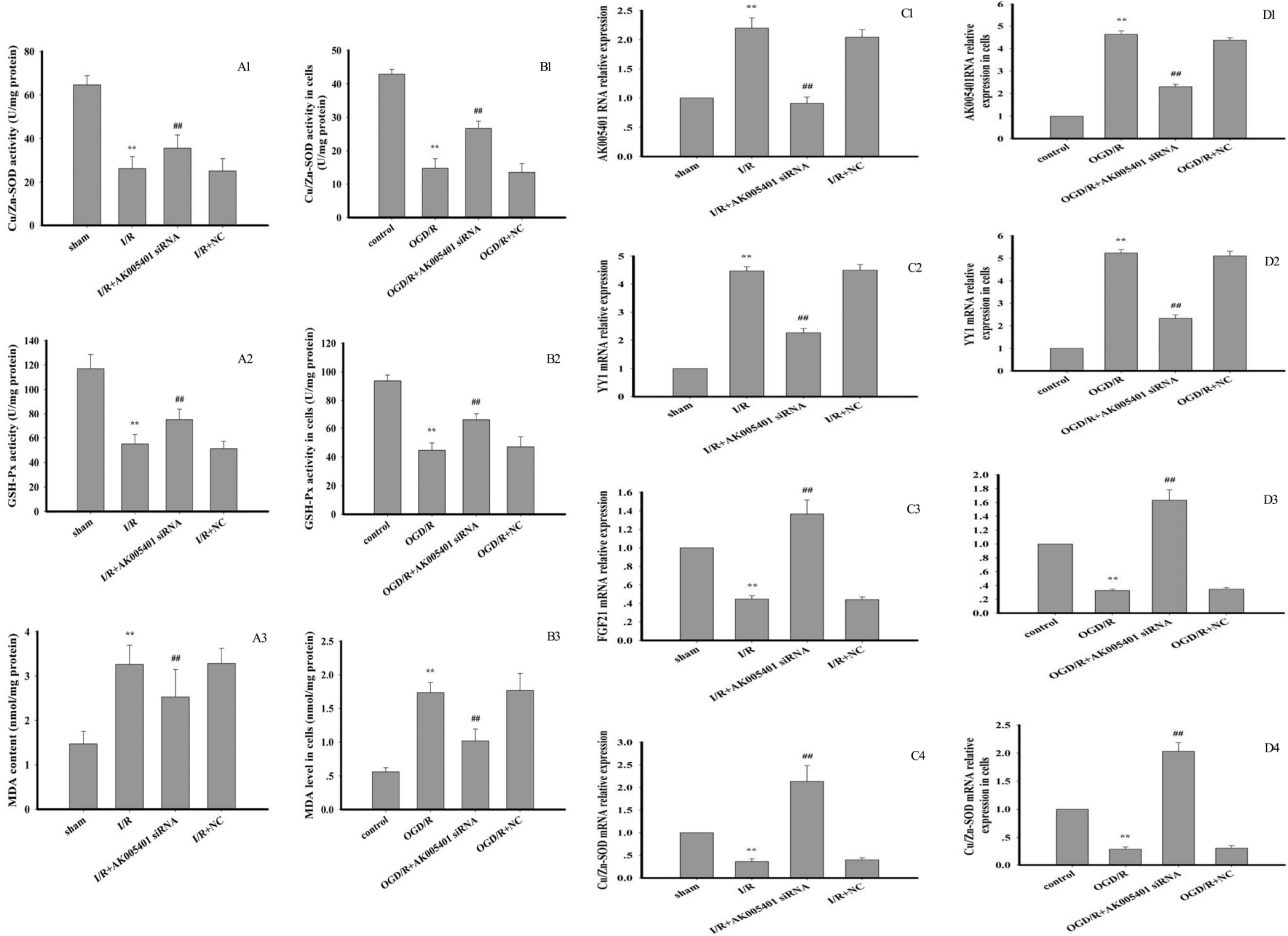
**Effect of AK005401 on antioxidant capacity and target genes expressions. Mice and HT22 cells were used to establish I/R model and OGD/R model, respectively.** The activities of CuZn SOD and GSH-Px and MDA level in tissue and cells were determined with spectrophotometrical method according to the procedure described by assay kit. (**A1–A3**) represent the activities of SOD and GSH-Px and MDA level in hippocampus tissue (n=10). (**B1–B3**) represent the activities of SOD and GSH-Px and MDA level in cells (n=8). Total RNA were isolated from vascular endotheliums and RAECs using Trizol (Invitrogen) according to the manufacturer’s instruction. cDNA was synthesized with PrimeScript reverse transcriptase (TaKaRa, Dalian, China) and oligo (dT) (20 bp) following the manufacturer’s instructions. Real-time PCR was performed using SYBR Premix Ex TaqTM II kit. Relative expressions of AK005401, YY1, FGF21, and CuZn SOD were calculated using the 2^−ΔΔCT^ method on a real-time PCR system. (**C1–C4**) represent the expression levels of AK005401, YY1, FGF21, and CuZn SOD in hippocampus tissue, respectively. (**D1–D4**) represent the expression levels of AK005401, YY1, FGF21, and CuZn SOD in HT22 cells, respectively. Data were presented as mean±SD (n = 3). One-way ANOVA test was used to determine statistical significance. ^**^P < 0.01 vs. normal group or control group, ^##^*P* < 0.01 vs. I/R group or OGD/R group.

As shown in Figure 4D–4F, OGD/R markedly promoted ROS generation (*P* < 0.01). However, treatment with AK005401 siRNA significantly inhibited OGD/R mediated ROS production (*P* < 0.01). As shown in Figures 4G, 5A1–5A3, 5B1–5B3, Relative to values observed in the sham and control groups, I/R and OGD/R markedly decreased the activity of Cu/Zn superoxide dismutase (Cu/Zn-SOD) and GSH-Px, stimulated ROS generation and increased levels of MDA in vitro and in vivo (*P* < 0.01), respectively. As shown in Figures 4G, 5A1–5A3, 5B1–5B3, AK005401 siRNA significantly increased the activity of antioxidant enzymes, inhibited ROS production, and decreased levels of MDA (*P* < 0.01).

### Effect of AK005401 on target gene expression

As shown in Figure 5C1–5C4, 5D1–5D4, relative to values observed in the sham and control groups, I/R and OGD/R significantly stimulated AK005401 expression (*P* < 0.01), which in turn directly enhanced the expression of YY1 and suppressed the expression of FGF21 and Cu/Zn-SOD (*P* < 0.01) in hippocampal tissues and cell cultures, respectively. However, these increases in YY1 expression were attenuated following treatment with AK005401 siRNA, which also produced significant increases in the expression of FGF21 and Cu/Zn-SOD in hippocampus tissues and HT22 cells (*P* < 0.01). We then assessed the effect of AK005401 overexpression on YY1/FGF21 expression. As shown in Figure 7B1–B3, compared to OGD/R group, overexpression of AK005401 significantly increased YY1 expression and reduced FGF21 expression (*P* < 0.05 or *P* < 0.01). Compared to OGD/R group, overexpression of YY1 obviously decreased FGF21 expression (*P* < 0.01). YY1 siRNA significantly decreased AK005401 expression and increased FGF21 expression compared to OGD/R+AK005401 group. Compared with OGD/R+YY1, overexpression of FGF21markedly reduced YY1expression (*P* < 0.05).

### Effect of AK005401 on target protein expression

**Figure 6:**
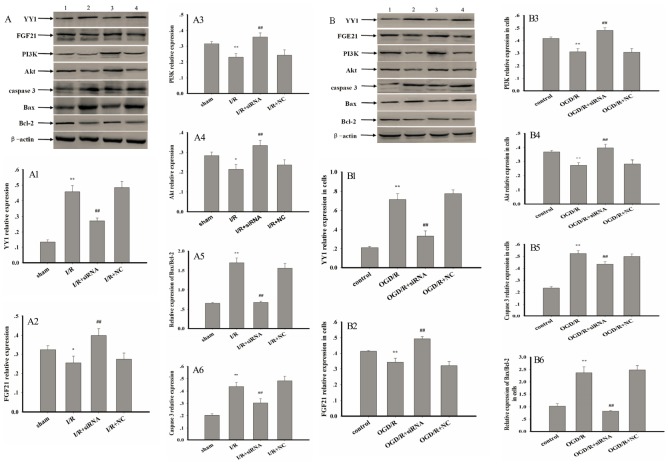
**Effect of AK005401 on target proteins expressions.** Mice and HT22 cells were used to establish I/R model and OGD/R model, respectively. The expression levels of YY1, FGF21, PI3K, Akt, caspase 3, Bcl-2, and Bax were visualized using chemiluminescence method. As shown in Figure 6, (**A**) represents Western blots in panel (1, 2, 3, and 4 represent sham, I/R, AK005401siRNA, and NC, respectively). (**A1–A6**) represent the relative expressions of YY1, FGF21, PI3K, Akt, caspase 3, and the relative ratio of Bax/Bcl-2 in hippocampus tissue, respectively. As shown in Figure 6-2, (**B**) represents Western blots in panel (1, 2, 3, and 4 represent control, OGD/R, AKsiRNA, and NC, respectively). (**B1–B6**) represent the relative expressions of YY1, FGF21, PI3K, Akt, caspase 3, and the relative ratio of Bax/Bcl-2 in HT22 cells, respectively. Data were presented as mean±SD (n = 3). One-way ANOVA test was used to determine statistical significance. ^*^P < 0.05 or ^**^P < 0.01 vs. sham group or control group, ^##^*P* < 0.01 vs. I/R group or OGD/R group.

**Figure 7:**
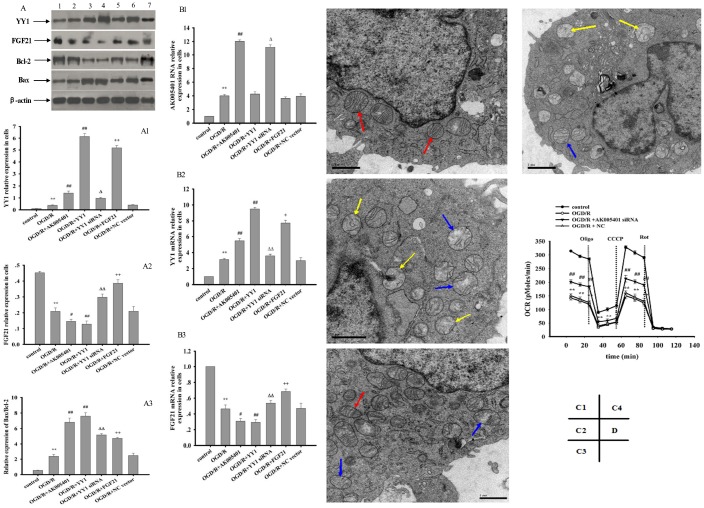
**Effect of overexpression of AK005401 and YY1 on the expressions of target genes and proteins and effect of AK005401 on mitochondrial structure and cell respiration.** HT22 cells were seeded in 96-well and 6-well plates and divided in seven groups for expression of target genes and proteins (control, OGD/R, OGD/R+AK005401, OGD/R+YY1, OGD/R+YY1 siRNA, OGD/R+FGF21 and OGD/R+NC vector) or four groups for cell respiration (control, OGD/R, AK005401siRNA, and NC). A represents Western blots in panel (1, 2, 3, 4,5,6 and 7 represent control, OGD/R, OGD/R+AK005401, OGD/R+YY1, OGD/R+YY1 siRNA, OGD/R+FGF21 and OGD/R+NC vector, respectively). (**A1–A3**) represent the relative expressions of YY1, FGF21and the relative ratio of Bax/Bcl-2 in cells, respectively. (**B1–B3**) represent represent the expression levels of AK005401, YY1 and FGF21 in HT22 cells, respectively. (**C1–C4**) represent mitochondria structure of control group, OGD/R group, AK005401siRNA group, and NC group, respectively, by using a transmission electron microscope (×20,000 magnification). Transmission electron microscope shows normal mitochondrial structure (red solid line arrows) and pathological mitochondria with irregular shape, as well as swollen (blue solid line arrow) and vesicular mitochondrial clusters (yellow solid line arrow). (**D**) represents mitochondrial oxygen consumption rate (OCR) of different groups. OCR was measured using an Oxygraph-2k system (n = 3 experiments per condition). Data were presented as mean±SD (n = 3). One-way ANOVA test was used to determine statistical significance. ^**^P < 0.01 vs. control group, ^#^*P* < 0.05 or ^##^*P* < 0.01 vs. OGD/R group, ^Δ^*P* < 0.05 or ^ΔΔ^*P* < 0.01 vs.OGD/R+AK005401 group, ^+^*P* < 0.05 or ^++^*P* < 0.01 vs.OGD/R+YY1 group

Relative to values observed in the sham and control groups, I/R and OGD/R significantly increased the expression of YY1, caspase 3, and Bax via upregulation of AK005401 expression in vitro and in vivo, respectively. Furthermore, I/R and OGD/R significantly suppressed the expression of FGF21, PI3K, Akt, and Bcl-2 in vitro and in vivo (Figure 6, *P* < 0.05 and *P* < 0.01), respectively. As shown in Figure 6, AK005401 siRNA significantly promoted the expression of FGF21, PI3K, Akt, and Bcl-2 and reduced the expression of YY1, caspase 3, and Bax by blocking the regulatory functions of AK005401 (*P* < 0.01). As shown in Figure 7A-A3, compared with OGD/R group, overexpression of AK005401and YY1 significantly increased YY1expression and the ratio of Bax/Bcl-2, decreased FGF21 expression (*P* < 0.05 and *P* < 0.01). YY1 siRNA remarkably decreased YY1expression and the ratio of Bax/Bcl-2, enhanced FGF21 expression compared to OGD/R+AK005401 group (*P* < 0.05 and *P* < 0.01). Overexpression of FGF21 significantly inhibited YY1expression and the ratio of Bax/Bcl-2 compared to OGD/R+YY1 group (*P* < 0.01).

### Effect of AK005401 on mitochondrial structure and cell respiration

In the control group, rod-shaped mitochondria were diffused throughout the cells and exhibited a normal structure (Figure 7C1). In OGD/R-treated cells, mitochondria exhibited pathological changes such as irregular and swollen shapes, damage to the mitochondrial ridge, fracturing of mitochondrial cristae, and severe vacuolization within mitochondria and vesicular mitochondrial clusters—especially those adjacent to the cell nucleus (Figure 7C2). Following treatment with AK005401 siRNA, an increased number of normal mitochondria were detected, and OGD/R-mediated mitochondrial injury was effectively alleviated (Figure 7C3). To examine the effects of AK005401 on cellular respiration, we measured the mitochondrial oxygen consumption rate (OCR) in HT22 cells. The OCR was markedly decreased in cells subjected to OGD/R (Figure 7D). Cells were first exposed to oligomycin, an ATP synthetase inhibitor, which caused an expected decrease in the OCR in all groups. To measure maximal mitochondrial respiratory capacity, cells were then treated with carbonyl cyanide m-chlorophenyl hydrazine (CCCP), which uncouples mitochondrial respiration. Cells normally increase the OCR in response to CCCP in order to maintain the proton gradient and mitochondrial function. However, cells exposed to OGD/R exhibited a smaller increase in OCR after CCCP treatment than that observed in control cells, suggesting that spare respiratory capacity in such cells is lower than that in controls. Administration of rotenone, a complex I inhibitor, also decreased the OCR. These data suggest that OGD/R impairs mitochondrial oxidative phosphorylation (OXPHOS) in HT22. Furthermore, AK005401 siRNA increased the OCR following CCCP treatment relative to that observed in the OGD/R group, suggesting that AK005401 decreases mitochondrial respiratory capacity.

## DISCUSSION

LncRNAs—which are defined as non-coding RNAs greater than 200 nucleotides in length—may act to upregulate biological processes in various disease states by directly or indirectly interacting with mRNA of the target gene. Such processes may ultimately lead to the destabilization and degradation of mRNA, modulation of apoptosis and invasion, and modification of chromatin [22–28]. In the present study, we demonstrated that I/R and OGD/R resulted in hippocampal damage accompanied by upregulation of AK005401. However, when AK005401 expression was blocked by siRNA, I/R- and OGD/R-mediated hippocampal damage was significantly attenuated. In addition, the data showed that overexpression of AK005401 significantly aggravated I/R and OGD/R mediated hippocampal neuron injury. These results indicated that AK005401 plays an important role in the exacerbation of ischemia-induced damage in the hippocampus.

ROS can function either as signaling molecules or as cellular toxicants. Excessive ROS can damage proteins, lipids, and DNA, which in turn leads to mitochondrial dysfunction, cell apoptosis, cell transformation, and genetic mutations that contribute to carcinogenesis [29]. Although mitochondrial and cellular oxidases synthesize a small amount of ROS under physiological conditions, ROS is quickly scavenged by antioxidant enzymes in order to maintain redox homeostasis. Previous studies have indicated that I/R can significantly enhance ROS generation and trigger oxidative stress by inhibiting the activity of antioxidant enzymes [30, 31], which were confirmed again in our experiment. Moreover, our findings further indicated that treatment with AK005401 siRNA significantly increased SOD and GSH-Px activity and remarkably decreased levels of MDA and ROS both *in vitro* and *in vivo*.

YY1 is a ubiquitous zinc finger transcription factor that can function as an activator or repressor of gene expression during embryogenesis, cellular proliferation, DNA replication [31], and cellular differentiation [32, 33]. Additional studies have demonstrated that YY1 suppresses CuZn-SOD gene expression and increases ROS levels by binding with negative regulatory elements in the CuZn-SOD promoter [21, 34]. In addition, YY1 can stimulate *Bax* expression, resulting in mitochondrial dysfunction and subsequent apoptosis [20]. In the present study, we observed that /R and OGD/R significantly increased levels of YY1 mRNA and protein and that such increases were effectively attenuated when AK005401 overexpression was inhibited using siRNA. Furthermore, our findings indicated that the activity of antioxidant enzymes decreased, while levels of ROS and MDA significantly increased, due to upregulation of YY1 expression under I/R conditions. Furthermore, I/R-mediated overexpression of AK005401 significantly increased cellular apoptosis and the expression of caspase 3 and Bax via upregulation of YY1, which were significantly attenuated by AK005401siRNA treatment. In addition, our findings demonstrated that YY1 siRNA effectively reduced apoptosis and the ratio of Bax/Bcl-2, increased cell viability, and then alleviated I/R induced-AK005401 overexpression mediated hippocampal injury.

Oxidative stress induces mitochondrial dysfunction, which is associated with various pathological conditions, including cardiovascular disease [35, 36]. Mitochondrial dysfunction enables electrons to escape from the mitochondrial respiratory chain, which then interact with oxygen molecules to form ROS [5]. ROS damage the mitochondrial membrane, facilitating the release of cytochrome C and promoting the activation of caspase 3, ultimately resulting in cellular apoptosis [11, 20]. In accordance with these findings, our results indicated that I/R and OGD/R caused structural damage in mitochondria (e.g., increases in mitochondrial volume and the formation of fractured cristae), resulting in mitochondrial dysfunction, reduced OCR, and increased ROS generation. Treatment with AK005401 siRNA substantially increased the number of healthy mitochondria, dramatically enhanced cellular respiratory function, and inhibited ROS production. The data showed that AK005401, as an important regulatory target, plays an important role in improving mitochondrial function and increasing antioxidant capacity.

Previous studies have indicated that activation of the PI3K/Akt signaling pathway is required to suppress cellular damage mediated by oxidative stress [37]. Further studies have demonstrated that activation of the PI3K/Akt signaling pathway improves mitochondrial function and increases antioxidant activity [38]. Yu et al reported that activation of PI3K and PI3K/Akt restored the balance between pro- and antiapoptotic Bcl-2 and Bax proteins and inhibited proapoptotic signaling events in mice [39]. FGF21, which is expressed in brown adipose tissue (BAT) and in the liver, is an endocrine hormone involved in the control of glucose homeostasis and lipid metabolism [40]. However, brain neurons also express FGF21, which plays a key role in conferring neuroprotective effects against glutamate challenge in rat cerebellar granular cells [41]. FGF21 plays a key role in alleviating MCAO-induced brain injury via activation of PI3K/Akt and inhibition of GSK-3β [42]. Research has indicated that FGF21 may activate *Sod2* and prevent ROS formation via the ERK pathway [43]. Planavila et al further demonstrated that FGF21 induces an antioxidant response by promoting the expression of certain antioxidant genes (*Ucp2*, *Sod2*) and preventing ROS formation [16]. In the present study, we observed that I/R or OGD/R significantly reduced FGF21 expression, thereby promoting ROS generation, enhancing the expression of caspase 3 and Bax, and triggering apoptosis. Moreover, our findings indicated that such alterations occurred via inactivation of the PI3K/Akt signaling pathway. Overexpression of FGF21 markedly decreased the ratio of Bax/Bcl-2 and reduced apoptosis, and then attenuated OGD/R mediated hippocampal neuron injury by blocking AK005401/YY1 signal pathway. AK005401 siRNA and YY1siRNA significantly increased antioxidant capacity and inhibited apoptosis, following which I/R-mediated hippocampal injury was attenuated by increases in FGF21 expression and activation of the PI3K/Akt pathway.

In conclusion, the findings of the present study indicated that AK005401 is an essential regulator in I/R-mediated hippocampal injury and that such injury is aggravated via downregulation of FGF21 expression, upregulation of YY1 expression, and activation of the PI3K/Akt pathway. These results show downregulation of AK005401 has potential application in cardiovascular diseases therapy.

## MATERIALS AND METHODS

### Reagents and antibodies

The siRNA oligos of AK005401 and YY1 and negative control RNA oligos (NC RNA oligos) were obtained from GenePharma (Shanghai Hi-Tech Park, Shanghai, China). Overexpression vectors of YY1, AK005401 and FGF21 were purchased from Genechem Co., Ltd. (Shanghai, China). Dulbecco’s modified Eagle medium (DMEM) and fetal bovine serum (FBS) were obtained from HyClone (Logan, UT, USA). Dimethyl sulfoxide (DMSO), antibodies, and 3-(4,5-dimethythiazol-2-yl)-2,5- diphenyl-tetrazolium bromide (MTT) were purchased from Sigma-Aldrich (St. Louis, MO, USA). Assay kits for ROS, superoxide dismutase (SOD), glutathione peroxidase (GSH-Px), malondialdehyde (MDA), and annexin V-FITC apoptosis were acquired from Nanjing Jiancheng Biology Engineering Institute (Nanjing, China). All other chemical reagents were of analytical grade.

### Animal model of global cerebral I/R

All animals were treated in accordance with the National Institutes of Health Guide for Care and Use of Laboratory Animals. Animal care and experimental procedures were approved by the Ethics Committee in Animal and Human Experimentation of Binzhou Medical University. Male C57Black/6 mice weighing 22±2 g were purchased from Jinan PengYue Laboratory Animal Breeding Co., Ltd (Jinan, China). All animals were housed in a temperature-controlled animal facility (23±2°C) under a 12-hour light/dark cycle, following which they were randomly divided into 4 groups: sham (n = 15), 20 min ischemia/24 h reperfusion (I/R; n = 15), 20 min ischemia/24 h reperfusion + AK005401 siRNA oligo (I/R+siRNA [100nM]; n = 15), 20 min ischemia/24 reperfusion + NC RNA oligo (I/R+ NC RNA; n = 15). Mice were anesthetized via an intraperitoneal injection of 10% chloral hydrate at a dose of 300 mg/kg (Sigma-Aldrich). AK005401 siRNAs with sense and antisense sequences of 5′- GCGUGUCUACACCGUCUAUTT-3′ and 5′- AUAGACGGUGUAGACAC GCTT-3′, respectively, were synthesized by GenePharma (Shanghai, China), who also provided a scrambled siRNA for use as a negative control. A total of 3 μl of AK005401 siRNA oligo, NC RNA oligo with lipofectamine or vehicle, was administered via intracerebroventricular injection after treatment with Bilateral common carotid artery occlusion (BCCAO). In addition, AK005401 overexpression vector group and NC vector group were added. A total of 4 μl of AK005401 over expression vector with lipofectamine or NC vector, were administered via intracerebroventricular injection before treatment with BCCAO. BCCAO was performed in accordance with methods described by Zhang et al [44]. Briefly, a ventral neck incision was made under sterile conditions, following which the right and left common carotid arteries were located lateral to the sternocleidomastoid and carefully separated. Cerebral ischemia was induced by clamping each of the arteries with two miniature artery clips (Yuyan Instruments Co., Ltd., Shanghai). Following 20 minutes of cerebral ischemia, the clips were removed from each artery to allow for the reperfusion of blood through the carotid arteries. Mice in the sham group underwent all surgical procedures except for artery occlusion. During the operation, body temperature was monitored using a rectal probe and maintained in the normal range (36.5-37.5ºC) using a heating lamp and a heating pad.

### Neurological tests

After 24 hours of reperfusion, all mice were subjected to a modified neurological examination designed to evaluate total motor deficits [45]. Briefly, mice were placed on a 10-20 cm horizontal screen (grid size: 0.2 × 0.2 cm), which was rotated from horizontal to vertical. The length of time each mouse remained on the vertical screen was recorded and scored as follows: 5 seconds = 1 point (maximum of 15 s). Mice were then placed at the center of a horizontal wooden rod (diameter: 1.5 cm), and the length of time each mouse remained on the rod was recorded and scored as follows: 10 seconds = 1 point (maximum of 30 s). Finally, mice were placed on a horizontal rope, and the length of time each mouse remained on the rope was recorded and scored as follows: 2 seconds = 1 point (maximum of 6 s). All neurological assessments were performed by an observer blinded to the mouse groups. A total motor score (TMS) was calculated based on the results of these three assessments (maximum score: 9 points).

### Hematoxylin and eosin (HE), cresyl violet and Fluoro-Jade B staining

Hippocampal pathomorphology was assessed via hematoxylin and eosin (HE) staining (Beyotime Institute of Biotechnology, Shanghai, China). After 24 hours of reperfusion, mice were anesthetized with 10% chloral hydrate (300 mg/kg intraperitoneally) and transcardially perfused with 4% phosphate-buffered paraformaldehyde, following a flush with 0.1 M phosphate-buffered saline (PBS). The brains were removed and post-fixed in 4% paraformaldehyde at 4°C overnight. The post-fixed brains were then removed, embedded in paraffin, cut into 4-μm-thick sections, stained with H&E or 0.5% cresyl violet, and visualized via light microscopy (LeiCA DM 5000B, Leica Microsystems, Wetzlar, Germany). Staining was then examined in the pyramidal neurons of the CA1 regions in the hippocampus (magnification ×200).

In addition, the dead cells in the CA1 region of hippocampus were quantitatively analyzed by using Fluoro-Jade B (FJB) staining. Briefly, the sections were immersed in a series of solutions, including 1% sodium hydroxide in 80% alcohol, 70% alcohol, 0.06% potassium permanganate, and 0.0004% FJB. Finally, the reacted sections were washed and placed on a slide warmer (approximately 50 °C) for 4 h. The digital images from 5 sections per animal were captured with an epifluorescent microscope F-JB^+^ cells were counted in a 250× 250μm square including the stratum pyramidale at the center of the CA1 area using an image analyzing system. Cell counts were obtained by averaging the counts.

### Cell cultures

HT22 cells were kindly provided by Professor Chunxiang Zhang (Department of Pharmacology, Rush University, USA). HT22 cells were cultured in high-glucose DMEM supplemented with 10% FBS and maintained at 37°C in a humidified mixture of air (95%) and CO_2_ (5%) (v/v). When monolayer cells had spread to more than 80% of the culture dish, cells were collected and seeded in a 96-well plate or 6-well plate (Costar, Cambridge, MA, USA) and divided into nine groups as follows: control, oxygen-glucose deprivation and reoxygenation (OGD/R), OGD/R +AK005401 siRNA, OGD/R+AK005401 (OGD/R+ AK005401 overexpression vector), OGD/R+YY1 siRNA (OGD/R + AK005401 overexpression vector +YY1 siRNA), OGD/R+YY1 (OGD/R+ YY1 overexpression vector), OGD/R+FGF21 (OGD/R + YY1 overexpression vector +FGF21 overexpression vector), OGD/R+NC (OGD/R+negative control RNA) and OGD/R+NC vector (OGD/R+negative control vector) groups. After a 24-hour treatment period, all groups other than the control group were cultured in glucose-free DMEM under hypoxic conditions (1% O_2_: 94% N_2_: 5% CO_2_) at 37°C for 4 hours. Thereafter, media were replaced with normal DMEM for all groups, and culturing continued for 20 hours to ensure reoxygenation under normoxic conditions (95% air: 5% CO_2_). An identical volume of vehicle was added at the same time in the control group.

### Cell viability and apoptosis assays

The thiazolyl blue tetrazolium bromide/3-(4,5-Dimethyl-2-thiazolyl)-2,5- diphenyl- 2H-tetrazolium bromide (MTT; Sigma-Aldrich) assay was used to determine mitochondrial dehydrogenase activity in cultured HT22 cells. The dark blue formazan crystals formed in the intact cells were solubilized with DMSO, and the absorbance was measured at 490 nm using a microplate reader (BioTek Synergy H4, USA). The results were expressed as a percentage of MTT reduction in the vehicle-treated control cells, for which an absorbance of 100% was assumed.

Cell apoptosis was detected via the terminal deoxyribonucleotidyl transferase-mediated dUTP-digoxigenin nick-end labeling (TUNEL) assay and annexin V-FITC/propidium iodide (PI) apoptosis assay methods, respectively. Briefly, HT22 cells were cultured and treated in accordance with the manufacturer’s instructions (Beyotime Institute of Biotechnology, China). Nuclei were labeled using 4′,6-diamidino-2- phenylindole (DAPI, Leagene Biotech, Beijing, China), and the active and apoptotic cells (blue and red, respectively) were visualized using an IX70-inverted fluorescence microscope (Olympus, Tokyo, Japan). TUNEL-positive apoptotic cells were expressed as a percentage of the total number of cells. Cells labeled via the annexin V-FITC/PI assay were then examined via flow cytometry (Beckman Coulter Inc., Brea, CA, USA), and the percentage of viable apoptotic cells was calculated. Independent experiments were repeated at least 3 times.

### Measurement of antioxidative enzyme activity and MDA levels

After 24 hours of reperfusion or reoxygenation, cerebral cortical tissue was removed, blotted and weighed, and then homogenized in 0.1 M phosphate buffer solution (pH 7.4). The homogenate was centrifuged at 3000 × *g* for 5 minutes at 4°C. The supernatants were used to spectrophotometrically determine MDA levels at 532 nm, Cu/Zn-SOD activity at 550 nm, and GSH-Px activity at 412 nm, in accordance with the manufacturer’s instructions (Nanjing Jiancheng Bioengineering Institute, Nanjing, PR China). Protein content was measured using a bicinchoninic acid (BCA) protein assay kit (Beijing Solarbio Science & Technology Co., Ltd., China).

### Measurement of total ROS generation

Following all experimental procedures, mice were euthanized via cervical dislocation. Hippocampal tissues were collected, weighed, and homogenized with saline on ice. The homogenate was centrifuged at 4000 × *g* for 15 min at 4°C. The supernatants were used to spectrophotometrically determine the level of ROS at 450 nm, in accordance with the manufacturer’s instructions (Shanghai Enzyme-linked Biotechnology Co., Ltd). Protein concentrations in each supernatant were detected using a BCA protein assay kit. HT22 cells were seeded in 6-well plates and treated for 24 hours as described above, following which they were co-cultured with 5 μM of 2,7-dichlorodihydrofluorescein diacetate (DCFH-DA) for 30 minutes in total darkness. The cells were collected and suspended in 500 μL of PBS for the measurement of ROS levels. The fluorescence intensity was immediately assayed via flow cytometry. The results were expressed as a percentage of the control value in untreated cells.

### Observation of mitochondrial structure

Treated HT22 cells were then fixed in 2.5% (v/v) glutaraldehyde and collected in a centrifuge tube, following which they were fixed in osmic acid, dehydrated via graded ethanol solutions, and embedded in epoxy resin. Embedded cells were sectioned into ultraslices, stained with uranyl acetate and lead citrate, and observed using a transmission electron microscope (Olympus, Tokyo, Japan).

### High-resolution respirometry

Cell respiratory function was analyzed via high-resolution respirometry, which was performed in a two-channel titration injection respirometer at 37°C (Oxygraph-2k, Oroboros, Innsbruck, Austria). Briefly, HepG2 cells were seeded in 60 mm dishes and treated in accordance with previously described methods [46]. Cell respiration was measured using the Oxygraph-2k system in accordance with the manufacturer’s instructions.

### Gene expression analysis

RNA was isolated from hippocampal tissues or HT22 cells using Trizol reagent (Invitrogen, USA), in accordance with the manufacturer’s instructions. The PrimeScript reverse transcription kit (Takara Bio Inc., Dalian, China) was then used to synthesize cDNA. Quantitative real-time polymerase chain reaction (qRT-PCR) was conducted using specific primers for AK005401, YY1, CuZn-SOD, and FGF21; the SYBR Premix Ex TaqTM II kit (Takara Bio Inc., Dalian, China); and an ABI 7000 RT PCR system. The RT PCR protocol was as follows: 95°C for 30 seconds, followed by 45 cycles at 95°C for 5 seconds, 60°C for 20 seconds to anneal, and 72°C for 15 seconds. We utilized *β*-actin as an internal control, and the relative expression levels of AK005401, YY1, CuZn-SOD, and FGF21 were calculated using the 2^-ΔΔCT^ method [47]. Each sample was analyzed in triplicate. The primers used for AK005401, YY1, FGF21, and CuZn-SOD were as follows: AK005401 forward primer: 5′-TGAGGAATGATTGACGAGGAA-3′; reverse primer: 5′-AACCTGGAGACAAGAACTGAT-3′; YY1 forward primer: 5′-TAACTT TGCTTGCGGTAGATT-3′; reverse primer: 5′-CTACAACTGAGCACCACTTTCT-3′; FGF21 forward primer: 5′- GGGGGTCTACCAAGCATACC-3′; reverse primer: 5′-TTGTAACCGTCCTCCAGCAG-3′; Cu/Zn-SOD forward primer: 5′- GAGCAT TCCATCATTGGCCG -3′; reverse primer: 5′- CGCAATCCCAATCACTCCAC -3′.

### Western blot analysis

HT22 cells and hippocampal tissue were lysed in cell lysis buffer (Beyotime, China) supplemented with protease and phosphatase inhibitors. Lysates were sonicated for 1 minute and centrifuged at 12,000 × *g* for 10 minutes at 4°C. Thirty micrograms of protein were then loaded to each lane and separated via SDS–PAGE containing 10% (w/v) polyacrylamide gel, following which the samples were transferred to polyvinylidene difluoride membranes. The membranes were incubated with the following primary antibodies at 4°C overnight: YY1, FGF21, PI3K, Akt, Bcl-2, Bax, and caspase 3. After incubation with secondary antibodies, protein bands were visualized and analyzed using the chemiluminescence method and then quantified via densitometry.

### Statistical analysis

Data are presented as the mean ±standard deviation (SD). The statistical analysis of the results was performed using the unpaired *t* test or investigations of one-way analysis of variance (ANOVA) as appropriate. All analyses were performed using SPSS 10.0 software (SPSS, Inc., San Rafael, CA, USA). *P* values < 0.05 were considered significant.
