# Correction for: Activation of AK005401 aggravates acute ischemia/reperfusion mediated hippocampal injury by directly targeting YY1/FGF21

**DOI:** 10.18632/aging.102458

**Published:** 2019-11-07

**Authors:** Hongzhi Wan, Ying Yang, Miao Li, Xin Liu, Yeying Sun, Kexin Wang, Chunxiang Zhang, Qingyin Zheng, Chaoyun Wang

**Affiliations:** 1 School of Pharmacy, Binzhou Medical University, Yantai, P. R. China; 2 Third class of senior high school, NO.2 Middle School of Yantai Shandong, Yantai, P. R. China; 3 Department of Biomedical Engineering and Department of Medicine, School of Medicine, University of Alabama at Birmingham, Birmingham, AL 35233, USA; 4 Department of Otolaryngology-Head and Neck Surgery, Case Western Reserve University, Cleveland, OH 44106, USA; 5 Hearing and Speech Institute, Binzhou Medical University, Yantai, P. R. China

**Keywords:** correction

**This article has been corrected:** The authors requested to remove affiliation 1 for Chunxiang Zhang; affiliation 3 is only affiliation of this author in the article. The authors also requested to update Author Contributions section. The authors declare that these corrections do not change the results or conclusions of this paper. The updated Author Contributions information is provided below.

**AUTHOR CONTRIBUTIONS**

C.Y. Wang, and Q.Y. Zheng designed the study, conducted the experiments, analyzed data, and wrote the manuscript. M. Li, Y. Yang, H.Z. Wan, X. Liu, K.X. Wang and Y.Y. Sun contributed experiments. Y. Y. Sun provided valuable materials. C.X. Zhang provided some key advices.

Original article: Aging. 2019; 11:5108–5123 . https://doi.org/10.18632/aging.102106

